# Supplementary material for: Novel and Potential Small Molecule Scaffolds as DYRK1A Inhibitors by Integrated Molecular Docking-Based Virtual Screening and Dynamics Simulation Study
Source: Molecules. 2022 Feb 9;27(4):1159. doi: 10.3390/molecules27041159 (PMC8875901; doi:10.3390/molecules27041159)
Supplement: Supplementary file 1 [file molecules-27-01159-s001.zip › molecules-1593456-supplementary.pdf]

Supporting Information

Novel and potential small molecule scaffolds as DYRK1A inhibitors by integrated molecular docking-based virtual screening and dynamics simulation study

Mir Mohammad Shahroz [1], Hemant Kumar Sharma [1,*], Abdulmalik S. A. Altamimi [2], Mubarak A. Alamri [2], Abuzer Ali [3], Amena Ali [4], Safar Alqahtani [2], Ali Altharawi [2], Alhumaidi B. Alabbas [2], Manal A. Alossaimi [2], Yassine Riadi [2], Ahmad Firoz [5], Obaid Afzal [2,*]

[1] Department of Pharmaceutical Chemistry, College of Pharmacy, Sri Satya Sai University of Technology and Medical Sciences, Sehore 466001, Madhya Pradesh, India; mirshahroz@gmail.com (M.M.S.); hkspharma@rediffmail.com (H.K.S.)

[2] Department of Pharmaceutical Chemistry, College of Pharmacy, Prince Sattam Bin Abdulaziz University, Al Kharj 11942, Saudi Arabia; as.altamimi@psau.edu.sa (A.S.A.A.); m.alamri@psau.edu.sa (M.A.A.); safar.alqahtani@psau.edu.sa (S.A.); altharawi@gmail.com (A.A.); ab.alabbas@psau.edu.sa (A.B.A.); m.alossaimi@psau.edu.sa (M.A.A.); y.riadi@psau.edu.sa (Y.R.); o.akram@psau.edu.sa (O.A.)

[3] Department of Pharmacognosy, College of Pharmacy, Taif University, P.O. Box 11099, Taif 21944, Saudi Arabia; abuali@tu.edu.sa (A.A.)

[4] Department of Pharmaceutical Chemistry, College of Pharmacy, Taif University, P.O. Box 11099, Taif 21944, Saudi Arabia; amrathore@tu.edu.sa (A.A.)

[5] Department of Biological Sciences, Faculty of Science, King Abdulaziz University, Jeddah 21589, Saudi Arabia; ahmadfirozbin@gmail.com (A.F.)

* Correspondence: hkspharma@rediffmail.com (H.K.S.); o.akram@psau.edu.sa (O.A.); Tel.: +919826512726 (H.K.S.); +966115886094 (O.A.)

Table of contents

S. No.	Content	Page No.
1.	**Table S1:** Thirty-five physicochemical and pharmacokinetic properties of six identified hit molecules, predicted by QikProp (Schrodinger) for orally active CNS drugs.	S3-S4
2.	**Table S2:** Toxicity profile of six identified hit molecules, predicted by ProTox-II.	S5
3.	**Table S3:** Comparison of kinase inhibition bioactivity, predicted by Swiss Target Prediction and molinspiration webservers, of the identified 6 hit ligands and the reported 3 inhibitors.	S6
4.	**Table S4:** Structural similarity analysis of six identified hits with the co-crystallized ligands of studied DYRK1A and related kinases by hierarchical clustering - distance matrix method (ChemMine Web Tools).	S7

Table S1. Thirty-five physicochemical and pharmacokinetic properties of six identified hit molecules, predicted by QikProp (Schrodinger) for orally active CNS drugs.

S. No.	Property	Description	Range for orally active CNS drugs QL	PL	PU	QU	21830	113352	159938	190037	218878	9539
1.	#stars	drug likeness penalty; the higher the value, the less drug-like the molecule	0	0	0	3	0	0	0	0	0	1
2.	#amine	no. of basic amines	0	1	1	2	0	0	0	0	0	0
3.	#amidine	no. of amidines groups	0	0	0	0	0	0	0	0	0	0
4.	#acid	no. of carboxylic acid groups	0	0	0	0	0	0	0	0	0	0
5.	#amide	no. of amides groups	0	0	0	1	0	0	0	0	0	0
6.	#rotor	no. of rotatable bonds (without CX3, alkene, amide, small ring)	0	3	6	8	4	2	2	3	2	0
7.	CNS	a qualitative CNS activity parameter	−2	0	1	2	0	-2	-1	-1	-1	-1
8.	dipole	computed dipole moment	0.67	1.1	3.9	8.9	3.86	1.03	3.25	2.48	3.25	7.92
9.	SASA	solvent accessible surface area	348	487	620	798	615.11	535.84	594.71	570.48	505.93	553.57
10.	FOSA	SASA on saturated carbon and attached hydrogen	16	178	314	464	227.55	117.13	66.19	6.7	241.95	74.59
11.	FISA	SASA on N, O, and H attached to heteroatoms	0	0	64	176	61.76	170.64	132.75	133.67	143.48	147.49
12.	PISA	π component of SASA	0	160	292	343	325.79	248.06	395.76	430.10	119.59	331.49
13.	WPSA	weakly polar component of the SASA (halogens, P, and S)	0	0	0	126	0	0	0	0	0	0
14.	volume	solvent accessible volume (Å^3)	492	830	1104	1388	1069.73	921.69	1052	935.91	847.97	966.91
15.	donorHB	estimated no. of hydrogen bonds that would be donated to the solvent water	0	0	1	3	1	2	0	1	1	2
16.	accptHB	estimated no. of hydrogen bonds that would be accepted from the solvent water	1	2.8	5.2	8.3	3.75	5	7	5	5	4.5
17.	glob	a globularity descriptor (1 for a sphere)	0.77	0.82	0.88	0.93	0.82	0.85	0.84	0.81	0.85	0.85
18.	QPpolrz	predicted polarizability (Å^3)	14	28	38	49	37.20	31.81	38.44	33.48	27.64	35.70
19.	QPlogPo/w	octanol−water logP	−0.16	2.5	4.7	6.0	4.37	1.96	2.26	2.63	1.67	2.68
20.	QPlogS	solubility in log(moles/liter)	−6.5	−4.6	−2.5	−0.42	-5.46	-3.87	-3.49	-4.33	-3.44	-4.69
21.	CIQPlogS	log of conformation-independent solubility	−6.3	−4.2	−2.3	0.36	-5.22	-4.11	-4.29	-3.93	-3.17	-4.78
22.	QPPCaco	apparent Caco-2 cell permeability	0	0	810	3269	2571.55	238.59	545.73	534.89	423.33	395.57
23.	QPlogBB	brain/blood partition coefficient	−1.2	−0.06	0.75	1.2	-0.28	-1.11	-0.80	-0.97	-0.87	-0.78
24.	QPPMDCK	predicted apparent MDCK cell permeability (nm/s)	0	0	634	5899	1373.16	105.11	257.07	251.55	195.36	181.55
25.	QPlogKhsa	prediction of binding to human serum albumin	−1	0.04	0.78	1.04	0.63	0.07	-0.28	0.06	-0.09	0.35

26.	Human Oral Absorption	Human oral absorption	2	3	3	3	3	3	3	3	3	3
27.	Percent Human Oral Absorption	Percent of human oral absorption	61	95	100	100	100	81.02	89.20	91.19	83.74	89.17
28.	PSA	van der Waals surface area of polar nitrogen and oxygen atoms	3.8	12	54	109	51.58	86.88	95.19	82.95	89.73	98.23
29.	#NandO	no. of N and O atoms	1	2	4	7	4	4	5	5	5	6
30.	Rule Of Five	no. of violations of Lipinski's rule of five	0	0	0	1	0	0	0	0	0	0
31.	Rule Of Three	no. of violations of Jorgensen's rule of three	0	0	0	1	0	0	0	0	0	0
32.	#in34	no. of atoms in three- or four-membered rings	0	0	0	0	0	0	0	0	0	0
33.	#in56	no. of atoms in five- or six-membered rings	5	11	17	24	19	18	20	17	13	16
34.	#noncon	no. of atoms not able to form conjugation in nonaromatic rings	0	0	4	10	2	1	0	0	2	0
35.	#nonHatm	no. of non-H atoms	8	19	25	30	24	22	26	21	19	24

Abbreviations: QL, qualifying lower limit; PL, preferred lower limit; QU, qualifying upper limit; PU, preferred upper limit.

Table S2. Toxicity profile of six identified hit molecules, predicted by ProTox-II.

S. No.	Classification	Property / Description	21830	11352	15938	19037	21878	9539
1.	Prediction Accuracy	---	68.07 %	67.38 %	68.07 %	69.26 %	67.38 %	54.26 %
2.	Predicted Toxicity Class (1-6)	Class 1: Most toxic, Class 6: Least toxic	4	4	4	4	4	1
3.	Predicted LD_{50} (mg/kg)	---	1000	1000	2000	1600	1600	1
4.	Organ toxicity	Hepatotoxicity	Inactive (0.91)	Inactive (0.69)	Inactive (0.50)	Active (0.59)	Inactive (0.63)	Active (0.56)
5.	Toxicity end points	Carcinogenicity	Inactive (0.62)	Inactive (0.53)	Active (0.64)	Active (0.72)	Inactive (0.53)	Active (0.55)
6.		Immunotoxicity	Inactive (0.97)	Inactive (0.97)	Inactive (0.77)	Inactive (0.99)	Inactive (0.93)	Inactive (0.99)
7.		Mutagenicity	Inactive (0.63)	Inactive (0.51)	Active (0.54)	Inactive (0.72)	Inactive (0.61)	Inactive (0.52)
8.		Cytotoxicity	Inactive (0.51)	Inactive (0.69)	Inactive (0.75)	Inactive (0.79)	Inactive (0.74)	Inactive (0.71)
9.	Tox21-Nuclear receptor signalling pathways	Aryl hydrocarbon Receptor (AhR)	Inactive (0.72)	Inactive (0.56)	Active (0.73)	Inactive (0.67)	Inactive (0.72)	Inactive (0.54)
10.		Androgen Receptor (AR)	Inactive (0.99)	Inactive (0.95)	Inactive (0.76)	Inactive (0.69)	Inactive (0.98)	Inactive (0.93)
11.		Androgen Receptor Ligand Binding Domain (AR-LBD)	Inactive (0.99)	Inactive (0.98)	Inactive (0.99)	Inactive (0.98)	Inactive (0.98)	Inactive (0.99)
12.		Aromatase	Inactive (0.92)	Inactive (0.91)	Inactive (0.79)	Inactive (0.88)	Inactive (0.87)	Inactive (0.85)
13.		Estrogen Receptor Alpha (ER)	Inactive (0.91)	Inactive (0.86)	Inactive (0.80)	Inactive (0.87)	Inactive (0.84)	Inactive (0.89)
14.		Estrogen Receptor Ligand Binding Domain (ER-LBD)	Inactive (0.98)	Inactive (0.96)	Inactive (0.93)	Inactive (0.97)	Inactive (0.94)	Inactive (0.98)
15.		Peroxisome Proliferator Activated Receptor Gamma (PPAR-Gamma)	Inactive (0.97)	Inactive (0.93)	Inactive (0.93)	Inactive (0.96)	Inactive (0.91)	Inactive (0.92)
16.	Tox21-Stress response pathways	Nuclear factor (erythroid-derived 2)-like 2/antioxidant responsive element (nrf2/ARE)	Inactive (0.97)	Inactive (0.91)	Inactive (0.98)	Inactive (0.58)	Inactive (0.87)	Inactive (0.95)
17.		Heat shock factor response element (HSE)	Inactive (0.97)	Inactive (0.91)	Inactive (0.98)	Inactive (0.58)	Inactive (0.87)	Inactive (0.95)
18.		Mitochondrial Membrane Potential (MMP)	Inactive (0.88)	Inactive (0.59)	Active (0.71)	Active (0.50)	Inactive (0.81)	Inactive (0.62)
19.		Phosphoprotein (Tumor Suppressor) p53	Inactive (0.92)	Inactive (0.75)	Inactive (0.80)	Inactive (0.53)	Inactive (0.90)	Inactive (0.86)
20.		ATPase family AAA domain-containing protein 5 (ATAD5)	Inactive (0.98)	Inactive (0.87)	Inactive (0.61)	Inactive (0.87)	Inactive (0.92)	Inactive (0.76)

Values in parenthesis indicates probability out of 1.

Table S3: Comparison of kinase inhibition bioactivity, predicted by Swiss Target Prediction and molinspiration webservers, of the identified 6 hit ligands and the reported 3 inhibitors.

MDPI (mol*Mall*) ID	Chemical Structures	Swiss target prediction (kinase inhibition, %)	Molinspiration bioactivity prediction (kinase inhibition score, range: -2.00 to +2.00)*
21830	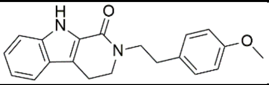	20%	+0.06
11352	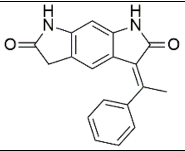	66.7%	+0.50
15938	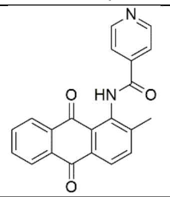	6.67%	+0.16
19037	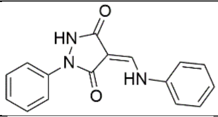	20%	+0.03
21878	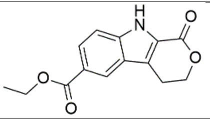	13.3%	-0.28
9539	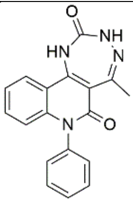	40%	-0.10
EHB	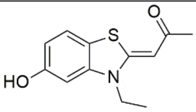	86.7%	-0.47
Harmine	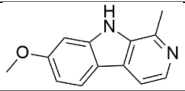	26.7%	+0.31
EGCG	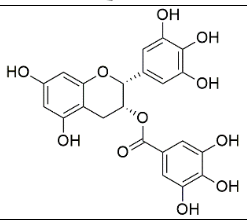	13.3%	+0.06

* If the bioactivity score is in between -1.00 to +1.00, then the molecule is said to be drug-like kinase inhibitor. More negative value indicate drug-like properties and more positive value indicates kinase inhibitory potential.

Table S4: Structural similarity analysis of six identified hits with the co-crystallized ligands of studied DYRK1A and related kinases by hierarchical clustering - distance matrix (ChemMine Web Tools).

Co-crystallized ligand-PDB ID (Kinase)	mol*Mall* ID (Z-scores*)					
	**21830**	**11352**	**15938**	**19037**	**21878**	**9539**
EHB-3ANQ (DYRK1A)	0.811	0.813	0.851	0.832	0.794	0.805
4E2-4YLK (DYRK1A)	0.812	0.623	0.751	0.713	0.792	0.623
QIV-5A3X (DYRK1A)	0.853	0.792	0.851	0.842	0.772	0.803
3RA-4AZE (DYRK1A)	0.682	0.643	0.712	0.612	0.731	0.682
D15-2WO6 (DYRK1A)	0.752	0.683	0.712	0.731	0.831	0.722
DYK-4D2S (DYRK1B)	0.764	0.796	0.807	0.814	0.846	0.807
IRB-3KVW (DYRK2)	0.835	0.677	0.774	0.736	0.848	0.699
3O0-3O0G (CDK5/p25)	0.807	0.737	0.768	0.729	0.856	0.777
9WG-5W4W (CK1)	0.786	0.707	0.798	0.795	0.828	0.697
DBQ-1Z57 (CLK1)	0.824	0.737	0.878	0.837	0.697	0.795
3RA-3RAW (CLK3)	0.682	0.643	0.712	0.612	0.731	0.682
B4K-5OY4 (GSK3beta)	0.807	0.705	0.818	0.795	0.818	0.728
STU-2PZY (MAPK2)	0.809	0.786	0.768	0.824	0.837	0.748
34I-3RTP (MAPK10)	0.725	0.723	0.808	0.779	0.795	0.778
8GX-5VUA (PIM1)	0.706	0.728	0.816	0.808	0.779	0.796
M77-5LCP (PKA)	0.745	0.857	0.908	0.868	0.839	0.856

Options used during analysis: Heatmap: distance matrix; Physicochemical Properties Heatmap: None; Linkage Method: complete; Properties Color and Display Values: Z-scores.

* Lower Z-score value (distance matrix) indicates, more similar the molecules, compared.
